# Milk metabolomic signatures associated with naturally occurring Staphylococcus chromogenes subclinical mastitis in early-lactation dairy cows

**DOI:** 10.14202/vetworld.2026.4191-4209

**Published:** 2026-09-22

**Authors:** Sillapapatra Sirituenlee, Suvaluk Seesupa, Sarinya Rerkyusuke

**Affiliations:** 1 Faculty of Veterinary Medicine, Khon Kaen University, Khon Kaen 40002, Thailand.; 2 Department of Veterinary Structural and Clinical Interventional Sciences, Faculty of Veterinary Medicine, Khon Kaen University, Khon Kaen 40002, Thailand.; 3 Department of Food Animal Clinical Sciences, Faculty of Veterinary Medicine, Khon Kaen University, Khon Kaen 40002, Thailand.; 4 Center for One Health and Animal Health, Faculty of Veterinary Medicine, Khon Kaen University, Khon Kaen 40002, Thailand.

**Keywords:** dairy cows, energy metabolism, intramammary infection, milk metabolomics, Staphylococcus chromogenes, subclinical mastitis, tricarboxylic acid cycle, untargeted metabolomics

## Abstract

**Background and Aim:**

Subclinical mastitis remains an important constraint on dairy production because it can impair udder health and milk quality without overt clinical signs. *Staphylococcus chromogenes* is a frequently isolated opportunistic pathogen associated with persistent subclinical intramammary infection; however, its pathogen-specific effects on the milk metabolome remain poorly characterized. This study aimed to characterize milk metabolomic alterations associated with naturally occurring *S. chromogenes* intramammary infection in early-lactation dairy cows and explore the underlying metabolic responses.

**Materials and Methods:**

A quarter-based comparative observational study was conducted on smallholder dairy farms in Khon Kaen, Thailand. Fifteen milk samples from nine early-lactation cows were analyzed, comprising eight *S. chromogenes*-infected and seven control quarters. Infection status was established using clinical assessment, the California Mastitis Test, somatic cell count (SCC), bacteriological culture, matrix-assisted laser desorption/ionization time-of-flight mass spectrometry, and *16S rRNA* gene sequencing. Milk metabolites were profiled using dual-polarity ultra-high-performance liquid chromatography-electrospray ionization-quadrupole time-of-flight tandem mass spectrometry. Multivariate discrimination was evaluated using orthogonal partial least squares-discriminant analysis (OPLS-DA), followed by univariate testing and metabolic pathway analysis.

**Results:**

SCC was significantly higher in infected than in control quarters (p < 0.05). Untargeted profiling detected 1,577 metabolites across positive and negative-ionization modes. OPLS-DA differentiated infected from control quarters, with R²X = 97.62%, R²Y = 96.89%, Q² = 55.28%, and permutation test p = 0.03. Five candidate discriminating metabolites were identified: cytidine 5′-diphosphate, citric acid, (S)-malate, and phenylacetylglycine showed increased abundance, whereas acetylcarnitine showed decreased abundance in infected quarters. Pathway analysis implicated the tricarboxylic acid cycle, glyoxylate and dicarboxylate metabolism, and phenylalanine metabolism at nominal significance; however, none remained significant after false discovery rate (FDR) correction.

**Conclusion:**

Naturally occurring *S. chromogenes* subclinical mastitis was associated with exploratory alterations in the milk metabolome, particularly involving energy, amino acid, and fatty-acid metabolism. The pathogen-specific design and naturally occurring infection model provide preliminary insight into host-pathogen metabolic interactions. However, given the small, partially paired sample and the absence of FDR-supported pathway associations, with only one of the five candidate metabolites surviving FDR correction, these findings should be considered hypothesis-generating. Larger independent cohorts with targeted quantitative validation are required before the identified metabolites can be considered diagnostic biomarkers.

## INTRODUCTION

Mastitis is one of the most prevalent and economically important diseases affecting the dairy industry, with substantial consequences for milk quality, milk production, and animal health [1]. Despite its economic importance, dairy farmers may underestimate mastitis-associated losses, estimated at approximately 5,000 Thai baht per affected dairy cow annually [2]. Mastitis is an inflammatory response of the mammary gland caused by physical injury or infection, with bacterial infection representing the predominant etiology [3]. Bacterial mastitis pathogens are conventionally classified as contagious, environmental, or opportunistic according to their reservoirs and modes of transmission. Subclinical mastitis is frequently associated with contagious and opportunistic pathogens [4–6] and typically occurs without visible abnormalities in the milk or overt clinical signs.

Among opportunistic pathogens, *Staphylococcus chromogenes* is an important coagulase-negative staphylococcus associated with bovine subclinical mastitis. Its ability to form biofilms may contribute to persistence within the mammary gland, complicate antimicrobial treatment, and promote chronic intramammary infection [7–10]. The reported prevalence of *S. chromogenes* varies considerably among geographical regions and study populations, ranging from approximately 12% to 38% in Thailand [11, 12], 20% to 22.1% in Southeast Asia [13–15], and 0.07% to 28.7% in studies conducted worldwide [16–19]. These observations underscore the epidemiological relevance of *S. chromogenes* while highlighting the need to better understand host responses associated with naturally occurring subclinical infection.

The milk metabolome reflects a complex interplay among mammary epithelial function, immune responses, physiological status, and environmental influences and can therefore provide insight into mammary gland health [20]. Comprehensive metabolite profiling offers an opportunity to characterize biochemical alterations associated with mastitis and to identify candidate metabolic signatures related to mammary inflammation and milk quality. This approach may be particularly informative for subclinical infections, in which metabolic disturbances can occur despite the absence of visible clinical abnormalities. Liquid chromatography-mass spectrometry (LC-MS)-based metabolomics provides high-resolution detection of diverse metabolites, and metabolomic approaches have increasingly been applied to investigate biochemical alterations associated with mastitis and other health disorders in ruminants [21, 22]. Previous LC-MS-based studies of intramammary infection have identified changes in metabolites such as asparagine, choline, glucose, and O-acetylcarnitine, indicating perturbations in amino acid, lipid, and energy metabolism [23].

Despite these advances, an important pathogen-specific knowledge gap remains. Most previous milk metabolomic studies of mastitis have investigated infections caused by major pathogens, mixed etiologies, or experimental challenge models, including those involving *Staphylococcus aureus*, *Escherichia coli*, and *Streptococcus* spp. [21, 23]. In contrast, the metabolic response associated specifically with naturally occurring *S. chromogenes* intramammary infection remains poorly characterized, despite the frequent detection of this organism in bovine subclinical mastitis in Thailand, Southeast Asia, and other geographical regions [11–19] and the continuing importance of mastitis in Thai dairy herds [24–26]. Pooling different mastitis pathogens may obscure pathogen-specific metabolic responses because organisms with different virulence characteristics and host interactions may not produce identical alterations in the milk metabolome. Furthermore, experimental challenge models may not fully represent the metabolic state associated with naturally occurring, persistent, low-grade subclinical infection. Consequently, the metabolic pathways associated specifically with naturally occurring *S. chromogenes* infection, particularly under tropical smallholder dairy-farming conditions, remain insufficiently defined. To our knowledge, no untargeted LC-MS-based characterization of the milk metabolomic signature of naturally occurring *S. chromogenes* intramammary infection under these conditions has been reported.

Therefore, this study aimed to characterize milk metabolomic alterations associated with naturally occurring *S. chromogenes* intramammary infection in early-lactation dairy cows and to explore the underlying host metabolic responses. Specifically, milk from infected and control quarters was subjected to dual-polarity ultra-high-performance liquid chromatography-quadrupole time-of-flight mass spectrometry (UHPLC-QTOF-MS) to characterize differences in metabolite profiles and identify metabolic pathways potentially associated with infection. The study was an exploratory, hypothesis-generating investigation to identify candidate metabolites and metabolic pathways for subsequent targeted validation, rather than to establish diagnostic biomarkers. By focusing on naturally occurring, pathogen-specific subclinical infection in smallholder dairy herds under tropical conditions, this study sought to provide a foundation for understanding the metabolic response to *S. chromogenes* and for future development of validated metabolite-based approaches to studying bovine udder health.

## MATERIALS AND METHODS

### Ethical approval

Milk sample collection and all procedures involving dairy cows were reviewed and approved by the Institutional Animal Care and Use Committee (IACUC) of Khon Kaen University, Thailand, under a protocol for a study of intramammary infection caused by *Streptococcus agalactiae* and *E. coli* (approval no. IACUC-KKU-152/66; approved January 17, 2024). The *S. chromogenes*-infected and control quarters analyzed in the present study were identified among the milk samples collected under this protocol. A subsequent protocol focusing on *S. chromogenes* and *Streptococcus uberis* mastitis was approved by the same committee (approval no. IACUC-KKU-(C)-93/69; approved September 21, 2026). All animal-related procedures were conducted in accordance with the guidelines for the ethical use of animals established by the National Research Council of Thailand. Written informed consent was obtained from all participating farm owners prior to enrollment. Owners were informed of the study objectives, the sampling procedures, the intended use and storage of milk and urine samples, and their right to withdraw any animal at any time without consequence. Signed consent forms are retained by the corresponding author. Access to the participating farms and permission to collect samples were granted directly by the farm owners under the same written consent; no additional institutional or cooperative permit was required for on-farm sampling. No personally identifiable information of farm owners was collected; farms and animals were assigned coded identifiers. Because written informed consent was obtained from every participating owner, no waiver of consent was sought or required. The study was observational and involved naturally occurring *S. chromogenes* intramammary infection in dairy cows managed under routine smallholder farm conditions; mastitis was not experimentally induced. Clinical examinations and collection of milk and urine samples were performed as part of the approved protocol. Only cows meeting the predefined eligibility criteria were enrolled, and animals with systemic illness, lameness, or medication use within 7 days before sampling were excluded. Milk sampling was performed at the mammary-quarter level using routine, minimally invasive procedures during normal milking, after which standard milking resumed. Diagnostic procedures included the strip-cup test, California Mastitis Test (CMT), somatic cell count (SCC), bacteriological culture, matrix-assisted laser desorption/ionization time-of-flight mass spectrometry (MALDI-TOF MS) and *16S rRNA* gene sequencing to confirm intramammary infection status. Sample collection and initial processing were conducted under the approved IACUC protocol. The handling and use of bacterial isolates complied with institutional registration requirements and applicable Thai regulations governing pathogens and animal toxins. Long-term storage and subsequent handling of the biological materials were approved by the Institutional Biosafety Committee of Khon Kaen University (approval no. 2564-11-0840), originally approved in 2021 and renewed on March 31, 2026. This approval remained continuously valid throughout the sample storage and handling period. The bacterial isolates recovered from milk samples and the milk aliquots reserved for metabolomic analysis were stored at −80°C in the Animal Health Technology Research Group Laboratory (Room VM3417), Faculty of Veterinary Medicine, Khon Kaen University, and will be retained for three years before disposal in accordance with institutional biosafety regulations. Residual milk and urine samples not required for these analyses were discarded after laboratory processing was completed. All procedures were carried out with consideration for animal welfare and in accordance with the approved institutional protocol.

### Study period and location

The study was conducted from June to October 2024, corresponding to the monsoon season in northeastern Thailand. Dairy cows were recruited from three smallholder farms affiliated with the Khon Kaen Dairy Cooperative in Mueang District, Khon Kaen Province, Thailand. Sample processing and primary laboratory procedures were performed at the Animal Health Technology Research Group Laboratory, Faculty of Veterinary Medicine, Khon Kaen University, Thailand. Species-level bacterial identification was conducted at the Clinical Laboratory Section, Srinagarind Hospital, Faculty of Medicine, Khon Kaen University. SCC analysis was performed at the Upper Northeastern Veterinary Research and Development Center, Department of Livestock Development, Tha Phra District, Khon Kaen Province. Metabolomic profiling was conducted at the National Phenome Institute, Khon Kaen University, Khon Kaen Province, Thailand.

### Study design

This study used a quarter-based comparative observational design to investigate milk metabolomic differences associated with naturally occurring *Staphylococcus chromogenes* subclinical intramammary infection. Milk samples were classified at the mammary-quarter level into *S. chromogenes*-infected and control groups using predefined clinical, cytological, bacteriological, proteomic, and molecular identification criteria. The final dataset comprised 15 mammary quarters from nine cows, including eight *S. chromogenes*-infected quarters and seven control quarters. Four cows contributed both a control quarter and at least one infected quarter, one cow (C08) contributed two infected quarters, and the remaining cows contributed one quarter each (Supplementary Table S1). Thus, the dataset was partially paired and quarter-level observations were not completely independent.

The required sample size was estimated using the resource equation method adapted from Arifin and Zahiruddin [27]:

*n* = DF/*k* + 1

where DF represents the error degrees of freedom, k represents the number of experimental groups and *n* represents the number of experimental units per group. Using an acceptable error DF range of 10–20 for two-group comparisons, the estimated requirement was 6–11 experimental units/group. Given the exploratory, hypothesis-generating nature of untargeted metabolomics, the study aimed to identify preliminary metabolic alterations and candidate metabolites for subsequent validation rather than establish diagnostic biomarkers [28–31].

Each cow had four mammary quarters; however, only quarters meeting the predefined case or control criteria were included. Quarters showing mixed bacterial growth, growth of pathogens other than *S. chromogenes*, subthreshold colony counts, or surplus control samples were excluded (Figure 1). Because four cows contributed both infected and control quarters, within-cow clustering could not be excluded. Moreover, the small number of cows and farms precluded reliable estimation of cow- and farm-level random effects; therefore, these effects were not incorporated into the statistical model. All participating farms belonged to the same dairy cooperative and followed comparable feeding, housing, and milking-management practices. Accordingly, the findings were considered exploratory and require validation in larger, independently sampled cohorts.

### Animals and eligibility criteria

The study population consisted of primiparous Holstein-Friesian dairy cows in early lactation (7–90 days in milk). Eligible cows showed no clinical signs of systemic disease or lameness and had received no medication during the 7 days preceding sampling. All enrolled animals maintained a body condition score (BCS) of 2.5–3.5 on a 5-point scale [32].

All cows were fed a diet with a concentrate-to-roughage ratio of 40:60. The concentrate contained 16%–17% crude protein and 60% total digestible nutrients, and straw constituted the primary roughage. Cows had *ad libitum* access to water and were milked twice daily under routine farm-management conditions. Before sample collection, urine was screened for ketone bodies using Cybow™ Urine Reagent Strips (DFI Co., Ltd., Gimhae, Gyeongsangnam-do, South Korea). The test is based on the sodium nitroprusside reaction and primarily detects acetoacetate, with weak sensitivity to acetone, but does not detect β-hydroxybutyrate. Urinary ketone testing was negative in all enrolled cows, indicating no detectable ketonuria at the time of sampling.

### Definition of infected and control quarters

Quarter-level infection status was established using a multistep diagnostic cascade incorporating clinical examination, strip-cup testing, the CMT, SCC, bacteriological culture, MALDI-TOF MS, and *16S rRNA* gene sequencing.

The experimental unit was the mammary quarter. Fifteen quarters were sampled from nine cows on three farms. Four cows contributed quarters to both groups, giving a partially paired design; three cows contributed control quarters only and two cows contributed infected quarters only. Quarter-level variables (CMT score, SCC, and the milk metabolome) were therefore analyzed at the level of the quarter (n = 15), whereas cow-level variables (days in milk, rectal temperature, and urinary ketone status) were recorded at the level of the animal (n = 9) and are reported descriptively. Only cows that were clinically healthy, afebrile (rectal temperature 38.0°C–39.0°C), nonlame, and negative on urinary ketone testing were eligible for inclusion.

Group assignment was based solely on bacteriological criteria. The infected group (n = 8) comprised quarters yielding pure growth of ≥10 colonies from 10 µL of milk (≥1,000 colony forming units [CFU]/mL), with the isolate identified as *S. chromogenes* by MALDI-TOF MS and confirmed by *16S rRNA* gene sequencing. All eight infected quarters fulfilled the criteria for subclinical mastitis, characterized by confirmed infection and elevated SCC in the absence of visible abnormalities in the milk or mammary gland. None of the infected quarters was classified as clinical mastitis (Supplementary Table S1).

 The control group (n = 7) comprised quarters from the same cohort with no bacterial growth on culture performed under identical conditions, a negative strip-cup test, milk that was nonviscous and white without fibrin or blood clots, and mammary tissue that was soft, nonedematous, and nonpainful on palpation. 

CMT score and SCC were recorded for every quarter at sampling but were not used for group assignment. 

### Milk sample collection and handling

Before milk collection, cows were rinsed twice with clean water. The udder was subsequently cleansed with water containing 200 ppm chlorine and dried with a clean cloth. Each mammary quarter was screened for clinical mastitis using the strip-cup test by visually examining foremilk on a dark-bottomed strainer for clots, flakes, watery appearance, or discoloration [33]. Subclinical mastitis screening was performed using the CMT according to National Mastitis Council (NMC) recommendations [34]. Reactions were scored on the five-level NMC ordinal scale as Negative, Trace, 1, 2, or 3, in ascending order of gel formation, and all observations were recorded. 

Milk samples were collected during the afternoon milking session. After the initial streams of milk were discarded to minimize teat-end contamination, foremilk was aseptically collected from each selected quarter into sterile plastic containers, after which routine milking resumed.

A total of 45 mL of milk was collected from each quarter and allocated as follows: 5 mL for metabolomic analysis, 10 mL for bacteriological culture and isolation, and 30 mL for SCC determination. Within 5 min of collection, samples were transferred to a portable cooler maintained at 4°C and transported to the Animal Health Technology Research Group Laboratory, Faculty of Veterinary Medicine, Khon Kaen University, within 60 min. Samples intended for primary bacterial isolation and identification were processed immediately upon arrival and within 2 h of collection.

### Primary bacterial isolation and identification

Milk samples were cultured according to standard NMC guidelines [34]. A 10-µL aliquot of well-mixed milk was streaked onto sheep blood agar plates (Quality Media and Supply Co., Ltd., Khon Kaen, Thailand) using a sterile calibrated loop. Plates were incubated aerobically at 37°C for 18–24 h, after which colony number, morphology, and hemolytic characteristics were recorded. Intramammary infection with coagulase-negative staphylococci (CNS) was defined as the growth of ≥10 colonies of a pure culture from a quarter-level milk sample in accordance with NMC guidelines [34].

Only samples yielding a single purified colony morphotype were retained for subsequent species identification. Species-level identification was performed at the Clinical Laboratory Section, Srinagarind Hospital, Faculty of Medicine, Khon Kaen University. Pure bacterial isolates were analyzed by MALDI-TOF MS using an Autoflex MALDI-TOF system (Bruker Daltonics GmbH & Co. KG, Bremen, Germany).

### Genotypic identification of *Staphylococcus chromogenes*

Genomic DNA was extracted from isolated *Staphylococcus* colonies using the GF-1 Bacterial DNA Extraction Kit (Vivantis, Selangor, Malaysia). Molecular confirmation of *S. chromogenes* was performed by amplification of the *16S rRNA* gene using the universal primers 518F (5′-CCAGCAGCCGCGGTAATACG-3′) and 800R (5′-TACCAGGGTATCT AATCC-3′), producing an amplicon of approximately 282 bp. The polymerase chain reaction (PCR) products were subsequently subjected to Sanger sequencing to determine the nucleotide sequence of the amplified region and confirm species identity.

The resulting nucleotide sequences and chromatograms were analyzed using Chromas software version 2.6.6 (Technelysium Pty Ltd., Queensland, Australia). Following manual editing, sequences were compared with reference sequences in the National Center for Biotechnology Information database using the Basic Local Alignment Search Tool for nucleotides (BLASTn; version 2.17.0).

Sequence analysis showed high similarity to the complete genome sequence of *S. chromogenes* strain BTM (GenBank accession no. CP133242.1). The sequence generated using primer 518F showed 99% query coverage and 99.65% identity, whereas the sequence generated using primer 800R showed 96% query coverage and 100% identity, confirming the isolates as *S. chromogenes*.

### SCC evaluation

For each sample, a 30-mL milk aliquot was refrigerated at 4°C and transported to the Upper Northeastern Veterinary Research and Development Center, Department of Livestock Development, Tha Phra District, Khon Kaen Province, Thailand. Samples were analyzed within 12 h of collection. SCC was determined using a Fossomatic™ 7 DC system (Foss Electric, Hilleroed, Denmark) according to the manufacturer's standardized procedures.

### Preparation of milk samples for metabolomic analysis

On arrival at the laboratory, the 5-mL milk aliquot designated for metabolomic analysis was subdivided into sterile 1.5-mL microcentrifuge tubes. Aliquots were initially stored at −20°C and transferred to −80°C within 15 min, where they were maintained for a maximum of 6 months. Before LC-MS analysis, frozen aliquots were transported on dry ice within 15 min to maintain the frozen state. Metabolomic profiling was performed at the National Phenome Institute, Khon Kaen University, Thailand.

For metabolite extraction, 200 µL of milk was transferred to a 1.5-mL microcentrifuge tube, and 600 µL of ice-cold methanol was added at a 3:1 methanol-to-milk ratio for protein precipitation. Samples were vortexed for 30 s and incubated at −20°C for 24 h. The samples were then centrifuged at 18,600 × *g* for 15 min at 4°C, and 200 µL of the resulting supernatant was transferred to a clean tube without disturbing the pellet. Subsequently, 200 µL of high-performance liquid chromatography-grade water and 600 µL of chloroform were added. The mixture was vortexed vigorously for 5 min and centrifuged at 13,700 × *g* for 15 min at 4°C. Finally, 150 µL of the upper aqueous phase was transferred to clean glass inserts in LC-MS vials, sealed, and maintained at 4°C until analysis.

Equal aliquots from all milk samples were pooled to generate a quality control (QC) sample according to Broadhurst *et al.* [35]. A 150-µL aliquot of the pooled QC sample was transferred to a high-performance liquid chromatography glass insert for LC-MS analysis. Internal standards were not added during extraction to minimize matrix-associated interference during untargeted profiling, following a modification of the protocol described by Wehrens *et al.* [36].

### UHPLC-ESI-QTOF-MS/MS analysis

Aqueous extracts were analyzed using a reverse-phase ultra-high-performance liquid chromatography-electrospray ionization-quadrupole time-of-flight tandem mass spectrometry (UHPLC-ESI-QTOF-MS/MS) system (Bruker Daltonics) in both positive (ESI+) and negative (ESI−) electrospray ionization (ESI) modes. Chromatographic separation was performed using a Bruker Intensity Solo high-performance liquid chromatography C18 column (2.1 × 100 mm, 2-µm particle size). MS/MS fragmentation was performed in data-dependent acquisition mode with automated precursor selection (AutoMS/MS; Bruker Daltonics) at mass-dependent collision energies of 15–40 eV. External calibration was performed before batch analysis, and automatic internal calibration using sodium formate cluster ions was applied during data acquisition to maintain mass accuracy. Comprehensive instrumental parameters are provided in Supplementary Table S2.

### QC and sequence validation

The sample injection sequence was fully randomized and bracketed by QC samples. Pooled QC samples, QC dilution series, extraction blanks, and reconstitution blanks were distributed throughout the analytical sequence to assess background contamination, analytical reproducibility, and signal drift. QC samples were analyzed at sequence initiation, after every five sample injections, and at sequence termination. Analytical reproducibility was evaluated using pooled QC injections, with a feature coefficient of variation (CV) <30% considered acceptable. Comprehensive QC performance metrics are provided in Supplementary Table S3.

### Data processing and metabolite annotation

Raw UHPLC-ESI-QTOF-MS/MS data were processed using MS-DIAL software version 5.5 for peak detection, deconvolution, alignment, and metabolite annotation. Processing parameters included an MS1 mass tolerance of 0.01 Da, MS2 mass tolerance of 0.025 Da, retention-time alignment tolerance of 0.1 min, minimum peak height of 1,000 amplitude units, signal-to-noise threshold of 10, and MS/MS similarity cutoff of 80%. Features detected in <50% of samples or exhibiting a QC relative standard deviation >30% were excluded. Additional processing parameters are provided in Supplementary Table S4. Positive- and negative-ionization datasets were processed independently and subsequently merged for downstream statistical analysis.

Metabolite annotation was performed by querying detected features against METLIN (accessed January 2025), the Human Metabolome Database (version 5.0), MassBank (release 2024.06), and LipidBlast (version 68). Annotation followed the five-level Level of Assignment (LoA) framework described by Vorkas *et al.* [37], in which progressively higher LoA levels reflect stronger analytical evidence supporting metabolite annotation. Comprehensive annotation parameters and spectral-matching criteria are provided in Supplementary Table S5. The annotation workflow incorporated accurate-mass matching against database entries, MS/MS spectral comparison with *in silico* fragmentation patterns, comparison with database or published spectra, and, where available, validation based on retention-time and molecular mass. The highest annotation confidence was assigned when MS/MS spectra were directly matched against authentic standards. In the present study, metabolite annotations reached LoA 2 or LoA 3; no metabolites were validated against authentic reference standards (LoA 4 or LoA 5).

### Blinding procedures

Bacterial culture, isolation, and purification were performed by the investigators, who were not blinded to sample origin or clinical status. Personnel conducting SCC analysis were blinded to individual animal identity and infection-group allocation because samples were processed using coded identifiers; however, farm origin remained identifiable. PCR products submitted for sequencing and bacterial identification were labeled with coded identifiers without disclosure of animal identity, farm origin, or infection-group allocation.

Metabolomic samples were submitted to the analytical facility with associated metadata, including group classification, for LC-MS data acquisition and downstream analysis. The injection order was randomized, with QC samples, QC dilution samples, and blanks interspersed throughout the sequence. Statistical analyses were subsequently performed using predefined analytical workflows and objective statistical criteria.

### Statistical analysis

Animal- and sample-level characteristics, including days in milk, rectal temperature, BCS, lameness score, clinical mastitis status, and urinary ketone levels, were evaluated using univariate statistical methods. Statistical assumptions, including normality, were assessed before analysis. Continuous variables were compared using Student's t-test or the Mann–Whitney U test according to data distribution. Categorical variables were analyzed using Fisher's exact test or the Fisher–Freeman–Halton exact test, as appropriate. SCC values were logarithmically transformed before analysis to satisfy normality assumptions. Assumption diagnostics are summarized in Supplementary Table S6. These analyses were performed using IBM SPSS Statistics version 28.0 (IBM Corp., Armonk, NY, USA). Because four cows contributed quarters to both groups, a restricted permutation test was additionally performed in which class labels were permuted only within cows. This constraint yields 24 distinct label assignments, and an exact p-value was obtained by complete enumeration.

For multivariate metabolomic analysis, spectral data were normalized using probabilistic quotient normalization (PQN) and subjected to Pareto scaling. Multivariate modeling was subsequently performed using orthogonal partial least squares-discriminant analysis (OPLS-DA) to evaluate group separation and identify candidate discriminatory variables. Model performance was assessed using seven-fold cross-validation, and potential overfitting was further evaluated using a 1,000-iteration permutation test based on classification accuracy. During seven-fold cross-validation, the dataset was randomly divided into seven folds; six folds were used for model training and the remaining fold for internal validation, with the procedure repeated until each fold had served as the validation set. Model goodness-of-fit and predictive performance were assessed using R²X, R²Y, and Q².

Candidate differential metabolites were prioritized using three criteria: a variable importance in projection (VIP) score >1 derived from the OPLS-DA model, an absolute correlation coefficient |p(corr)[1]| ≥0.6 in the S-plot, and a nominal p < 0.05 by Student's t-test. Given the exploratory nature of the analysis, candidate metabolite findings were interpreted as hypothesis-generating rather than confirmatory. Hierarchical clustering analysis was performed using MetaboAnalyst 6.0 to visualize relative abundance patterns of the selected metabolites across samples. The normalized intensity data were autoscaled before clustering. Hierarchical clustering was performed using Euclidean distance and Ward’s linkage method, and the resulting abundance patterns were visualized as a heatmap. This analysis was used for exploratory visualization and not for formal hypothesis testing.

Metabolic pathway analysis (MetPA) was conducted using MetaboAnalyst version 6.0, with *Bos taurus* specified as the reference organism. Pathway enrichment was evaluated using the hypergeometric test, and pathway topology was assessed using relative-betweenness centrality. Metabolite annotations were mapped to Kyoto Encyclopedia of Genes and Genomes (KEGG) identifiers before pathway analysis, and only metabolites successfully mapped to pathways within the *B. taurus* KEGG library were included. False discovery rate (FDR) correction was performed using the Benjamini–Hochberg procedure.

The complete workflow from sample selection and collection through metabolomic profiling and statistical analysis is summarized in Figure 1.

## RESULTS

### Sample characteristics

The dairy cows had a mean lactation period of 49.6 days (range, 16–81 days), and none were pregnant. The median BCS was 3.5 (interquartile range, 2.5–3.5). No cows exhibited clinical mastitis or lameness. Accordingly, all animals had a lameness score of 0, and all eight infected quarters were classified as subclinical mastitis. Urinary ketone testing was negative in all cows. No significant differences in the evaluated characteristics were observed between the control and *S. chromogenes*-infected groups (Table 1).

### Relationship between SCC and infection status

Log-transformed SCC and CMT results are presented in Table 2. Log_10_ SCC was significantly higher in *S. chromogenes*-infected quarters than in control quarters (5.06 ± 0.68 vs. 4.10 ± 0.60, respectively; p = 0.01). The distribution of CMT scores did not differ significantly between groups (p = 0.06). Among infected quarters, six of eight (75.00%) had a CMT result of Trace and two (25.00%) had a score of 1. Among control quarters, four (57.14%) were Negative, two (28.57%) had a Trace result, and one (14.29%) had a score of 1.

**Figure 1:**
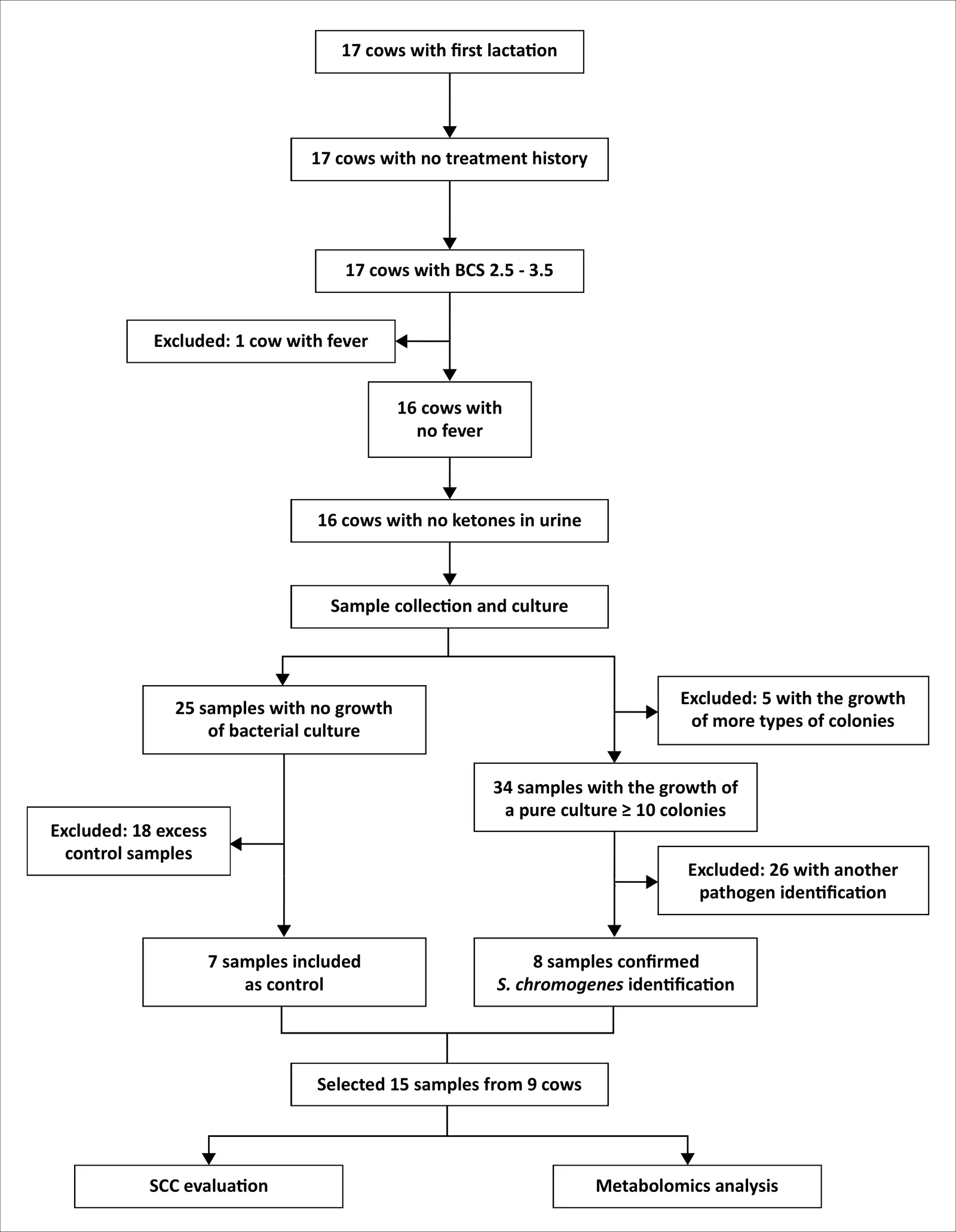
Schematic flowchart illustrating the experimental workflow from sample selection through statistical data analysis.

**Table 1:** Characteristics of the study samples according to udder health status

**Characteristics**	**Control (n = 7)**	***S. *** ***chromogenes*** ** (n = 8)**	**p-value**
Days in milk, mean ± SD	48.86 ± 25.76	50.25 ± 24.85	0.99
Rectal temperature (°C), mean ± SD	38.36 ± 0.41	38.15 ± 0.28	0.27
BCS			0.24
2.5	1 (14.29%)	4 (50.00%)	
3.0	2 (28.57%)	0 (0.00%)	
3.5	4 (57.14%)	4 (50.00%)	
Lameness (All cows, n = 9)	0 (0.00%)	0 (0.00%)	NA
Clinical mastitis (All cows, n = 9)	0 (0.00%)	0 (0.00%)	NA
Urinary ketones (All cows, n = 9)	0 (0.00%)	0 (0.00%)	NA

BCS = Body condition score; NA = Not applicable; SD = Standard deviation.

### Metabolite analysis

In ESI− mode, 465 metabolites were annotated, including 60 classified as LoA 3 and 405 as LoA 2. In ESI+ mode, 1,112 metabolites were annotated, including 72 classified as LoA 3 and 1,040 as LoA 2 (Supplementary Table S7). The mean CV across the seven QC replicates was 29.67% (Supplementary Table S3).

**Table 2:** CMT and log-transformed SCC according to udder health status

**Variable**	**Control (n = 7)**	***S. *** ***chromogenes*** ** (n = 8)**	**p-value**
Log₁₀ SCC (cells/mL), mean ± SD	4.10 ± 0.60	5.06 ± 0.68	0.01
CMT			0.06
Negative	4 (57.14%)	0 (0.00%)	
Trace	2 (28.57%)	6 (75.00%)	
1	1 (14.29%)	2 (25.00%)	
2	0 (0.00%)	0 (0.00%)	

CMT = California Mastitis Test; SCC = Somatic cell count; SD = Standard deviation. CMT reactions were scored on the five-level NMC scale (Negative, Trace, 1, 2, 3) in ascending order; no quarter scored 2 or 3. NMC = National Mastitis Council.

OPLS-DA was performed to evaluate metabolomic separation between the control and *S. chromogenes*-infected groups. The model demonstrated separation between the groups (Figure 2), with R²X = 97.62%, R²Y = 96.89%, and Q² = 55.28%, indicating high goodness-of-fit and moderate predictive performance. Permutation testing with 1,000 iterations yielded p = 0.03 (Figure 3). Under within-cow restricted permutation, the observed model ranked first among all 24 admissible label assignments (exact p = 0.042).

**Figure 2:**
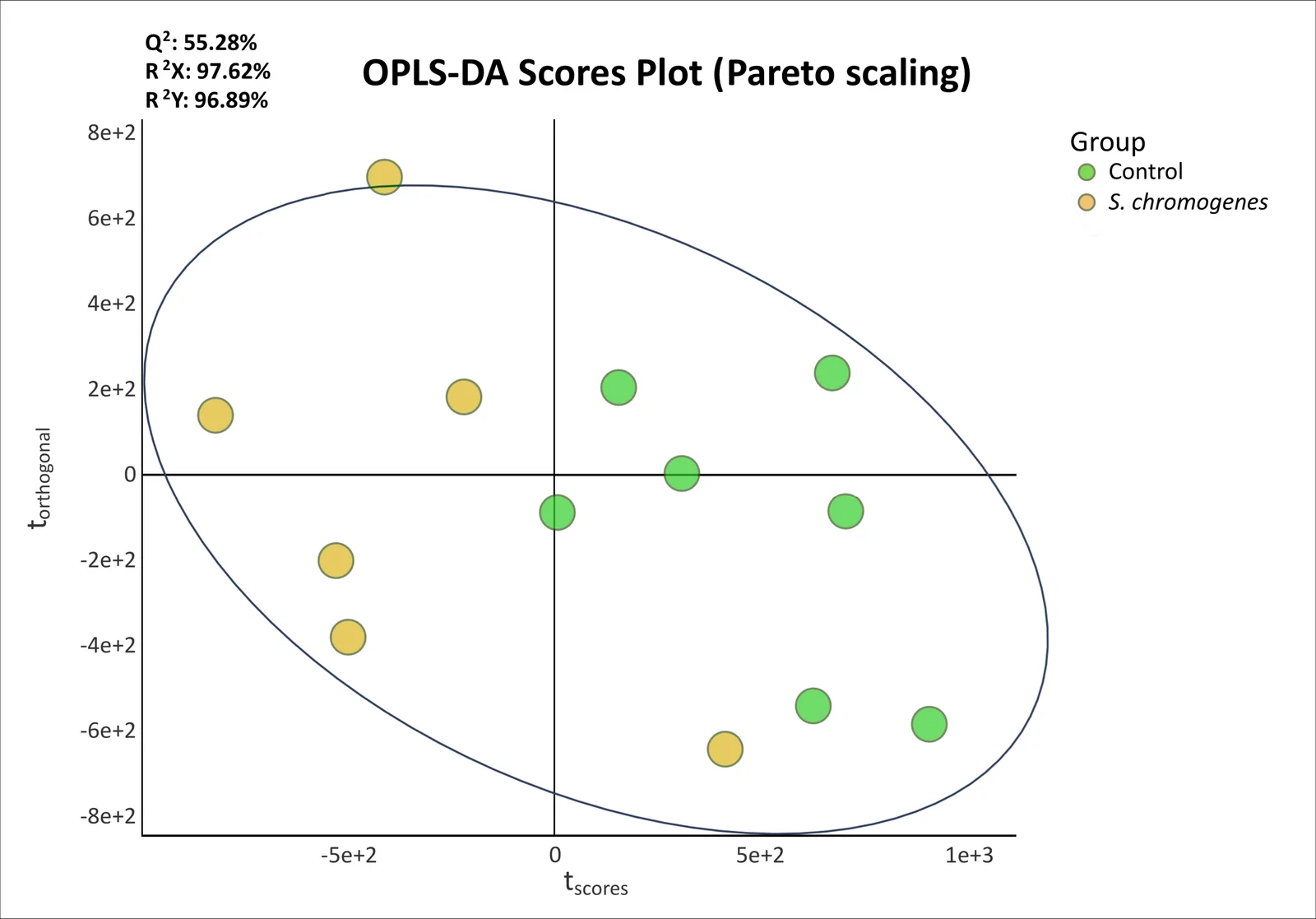
OPLS-DA score plot comparing milk metabolomic profiles between control and *Staphylococcus chromogenes*-infected quarters following PQN and Pareto scaling. Control and *S. chromogenes*-infected samples are shown in green and yellow, respectively. OPLS-DA = Orthogonal partial least squares-discriminant analysis; PQN = Probabilistic quotient normalization.

**Figure 3:**
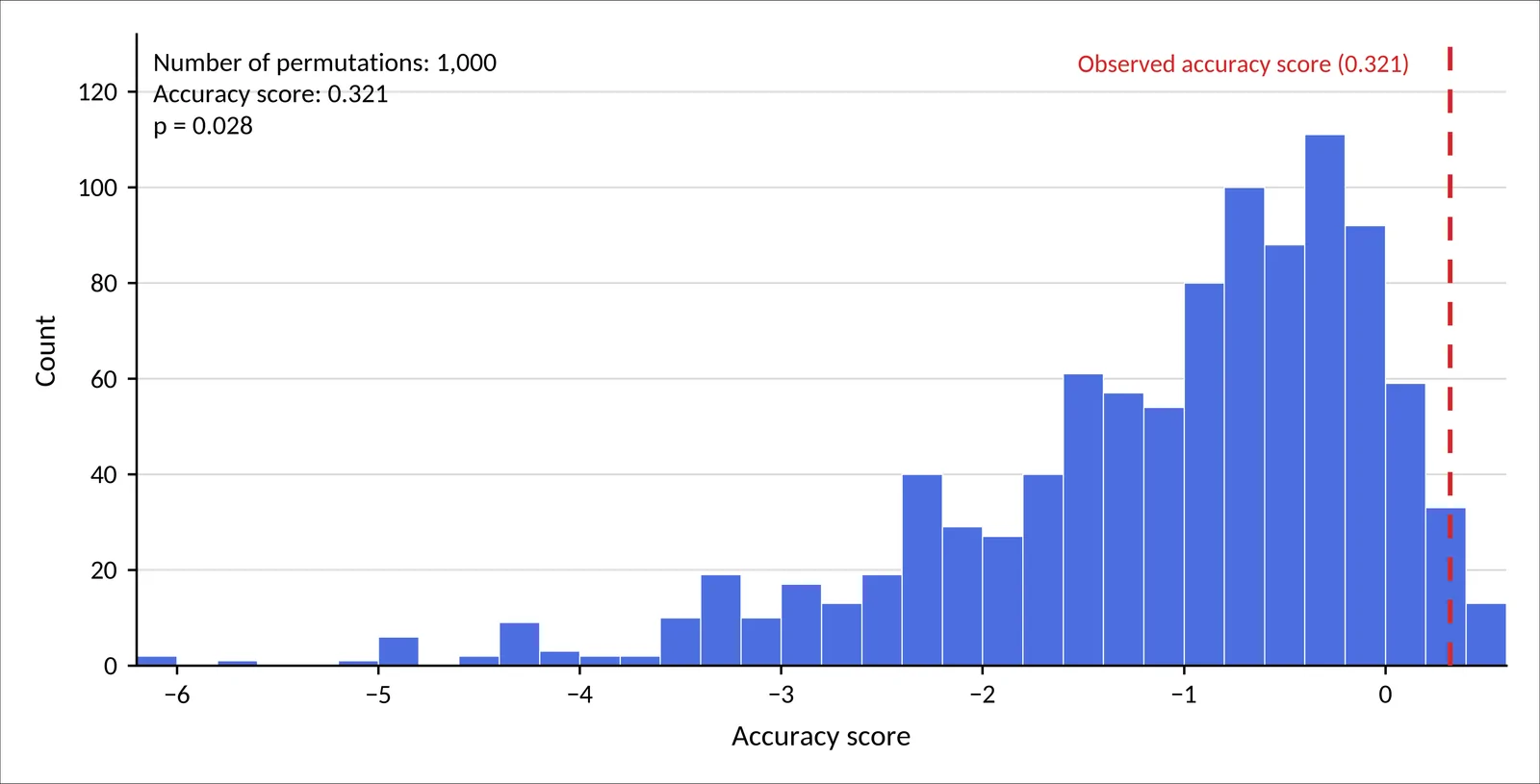
Permutation test (n = 1,000) for validation of the OPLS-DA model comparing control and *Staphylococcus chromogenes*-infected quarters. The histogram represents the distribution of accuracy scores obtained after random permutation of class labels, whereas the red dashed line represents the observed accuracy score of the original model (0.321). Permutation testing yielded p = 0.03. OPLS-DA = Orthogonal partial least squares-discriminant analysis.

The S-plot and VIP scores were examined to identify metabolites contributing most strongly to group discrimination (Figure 4). Five metabolites met all prioritization criteria: cytidine 5′-diphosphate (VIP = 1.98), citric acid (VIP = 1.50), (S)-malate (VIP = 1.39), acetylcarnitine (VIP = 1.38), and phenylacetylglycine (VIP = 1.37). Cytidine 5′-diphosphate, citric acid, (S)-malate, and phenylacetylglycine showed greater abundance in *S. chromogenes*-infected quarters, whereas acetylcarnitine showed lower abundance than in controls.

### Identification of discriminant metabolites

Five endogenous metabolites were identified as major contributors to group discrimination: citric acid, (S)-malate, cytidine 5′-diphosphate, acetylcarnitine, and phenylacetylglycine (Supplementary Table S8). All five candidates had VIP >1, |p(corr)[1]| ≥0.6, and p < 0.05. Their relative abundances in control and *S. chromogenes*-infected quarters are shown in Figure 5.

**Figure 4:**
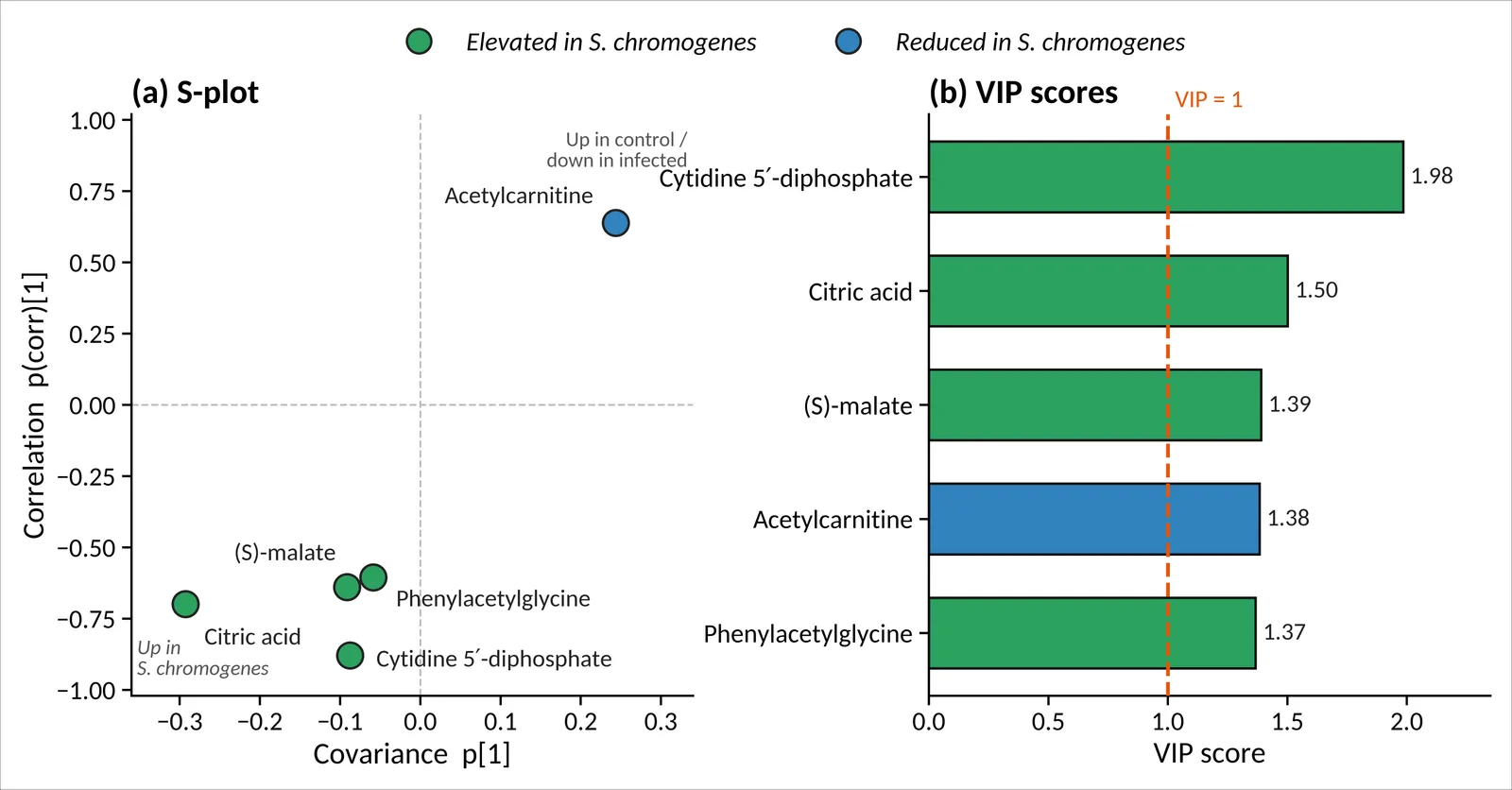
OPLS-DA of milk metabolites differentiating control and *Staphylococcus chromogenes*-infected quarters. (a) S-plot showing the relationship between covariance p[1] and correlation p(corr)[1]. Green points represent metabolites with greater abundance in infected quarters, including citric acid, (S)-malate, phenylacetylglycine, and cytidine 5′-diphosphate, whereas the blue point represents acetylcarnitine, which showed lower abundance in infected quarters. (b) VIP scores for the five candidate metabolites. The dashed line represents the VIP = 1 threshold. VIP scores were 1.98 for cytidine 5′-diphosphate, 1.50 for citric acid, 1.39 for (S)-malate, 1.38 for acetylcarnitine, and 1.37 for phenylacetylglycine. OPLS-DA = Orthogonal partial least squares-discriminant analysis; VIP = Variable importance in projection.

**Figure 5:**
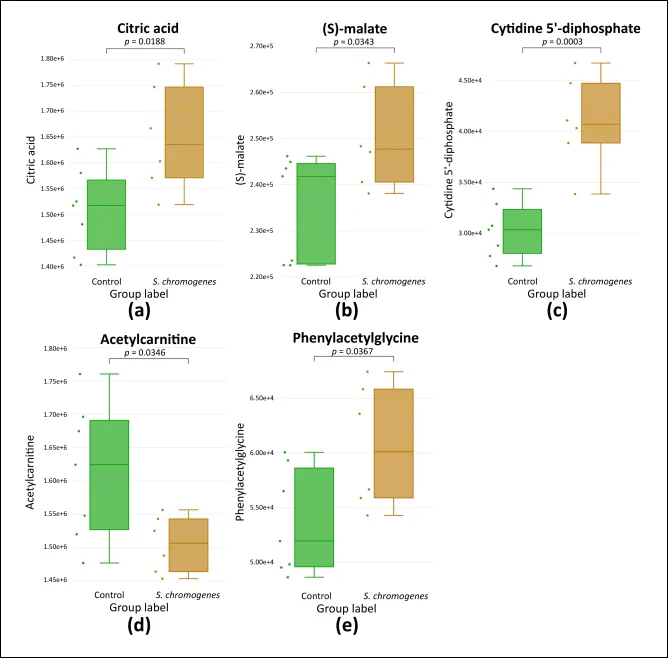
Box plots showing the relative abundances of candidate metabolites in control and *Staphylococcus chromogenes*-infected quarters. Nominal p-values were obtained using univariate t-tests. (a) Citric acid; (b) (S)-malate; (c) cytidine 5′-diphosphate; (d) acetylcarnitine; and (e) phenylacetylglycine.

At the nominal significance level, citric acid (p = 0.02), (S)-malate (p = 0.03), cytidine 5′-diphosphate (p < 0.01), and phenylacetylglycine (p = 0.04) showed greater abundance in *S. chromogenes*-infected quarters, whereas acetylcarnitine showed lower abundance (p = 0.03) (Table 3). After Benjamini–Hochberg correction across all detected features, only cytidine 5′-diphosphate remained significant (FDR = 0.0158); the corresponding FDR-adjusted p-values for citric acid, (S)-malate, acetylcarnitine, and phenylacetylglycine were 0.1384, 0.1952, 0.1961, and 0.2035, respectively, and these four should therefore be regarded as nominal candidate signals.

**Table 3:** Candidate discriminating metabolites between control and S. chromogenes-infected quarters

**Metabolite**	**Molecular formula**	**Ionization mode**	**Adduct type**	**m/z**	**Retention-time (min)**	**LoA**	**p-value**	**FDR-adjusted p-value**	**Trend**	**Log₂ FC**
Citric acid	C₆H₈O₇	Positive	[M+Na]⁺	215.01429	1.51	3	0.02	0.1384	↑	0.13
(S)-Malate	C₄H₆O₅	Negative	[M−H]⁻	133.01271	1.26	3	0.03	0.1952	↑	0.09
Cytidine 5′-diphosphate	C₉H₁₅N₃O₁₁P₂	Positive	[M+H]⁺	404.03361	1.51	2	<0.01	0.0158	↑	0.44
Acetylcarnitine	C₉H_18_NO₄	Positive	[M]⁺	204.12178	1.25	3	0.03	0.1961	↓	−0.10
Phenylacetylglycine	C₁₀H₁₁NO₃	Negative	[M–H]⁻	192.06419	7.01	3	0.04	0.2035	↑	0.18

FC = Fold change; FDR = False discovery rate; LoA = Level of Assignment; m/z = Mass-to-charge ratio.

Hierarchical clustering provided an exploratory visualization of relative abundance patterns of candidate metabolites between control and *S. chromogenes*-infected quarters (Figure 6).

**Figure 6:**
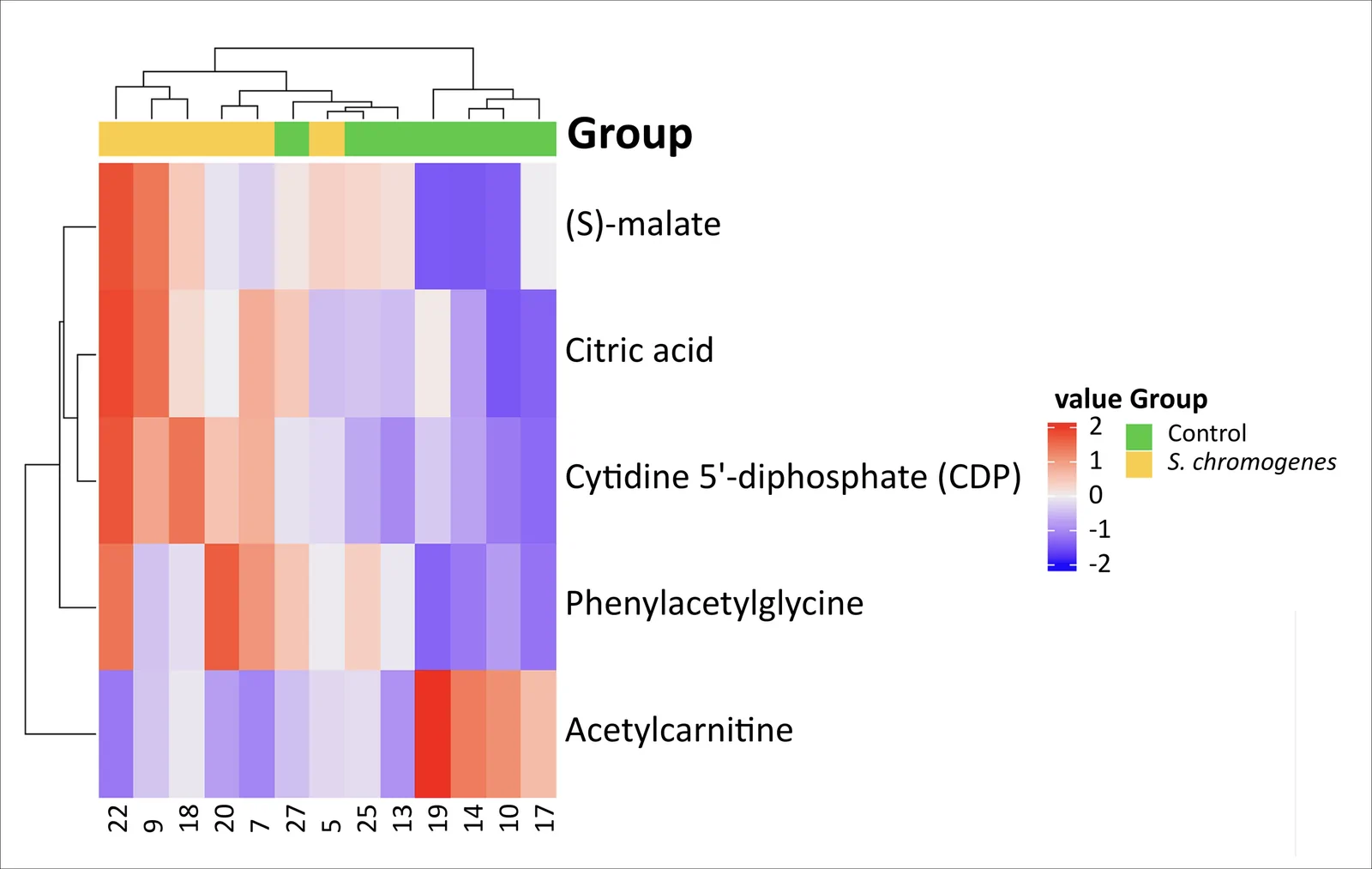
Hierarchical clustering heatmap illustrating relative abundance patterns of candidate milk metabolites between control and *Staphylococcus chromogenes*-infected quarters.

### MetPA

MetPA was performed using candidate metabolites mapped to KEGG identifiers. Although five candidate metabolites were identified, only four, citric acid, (S)-malate, cytidine 5′-diphosphate, and phenylacetylglycine, were included in the pathway analysis. Acetylcarnitine was excluded because no corresponding annotated node was available in the *Bos taurus* KEGG pathway maps. These four metabolites mapped to six metabolic pathways (Figure 7 and Table 4).

At the nominal significance level, the citrate cycle (tricarboxylic acid [TCA] cycle; p < 0.01), glyoxylate and dicarboxylate metabolism (p < 0.01), and phenylalanine metabolism (p = 0.03) were enriched. However, none of the six pathways remained statistically significant after FDR correction. The lowest FDR values were observed for the TCA cycle (FDR = 0.07) and glyoxylate and dicarboxylate metabolism (FDR = 0.09).

**Figure 7:**
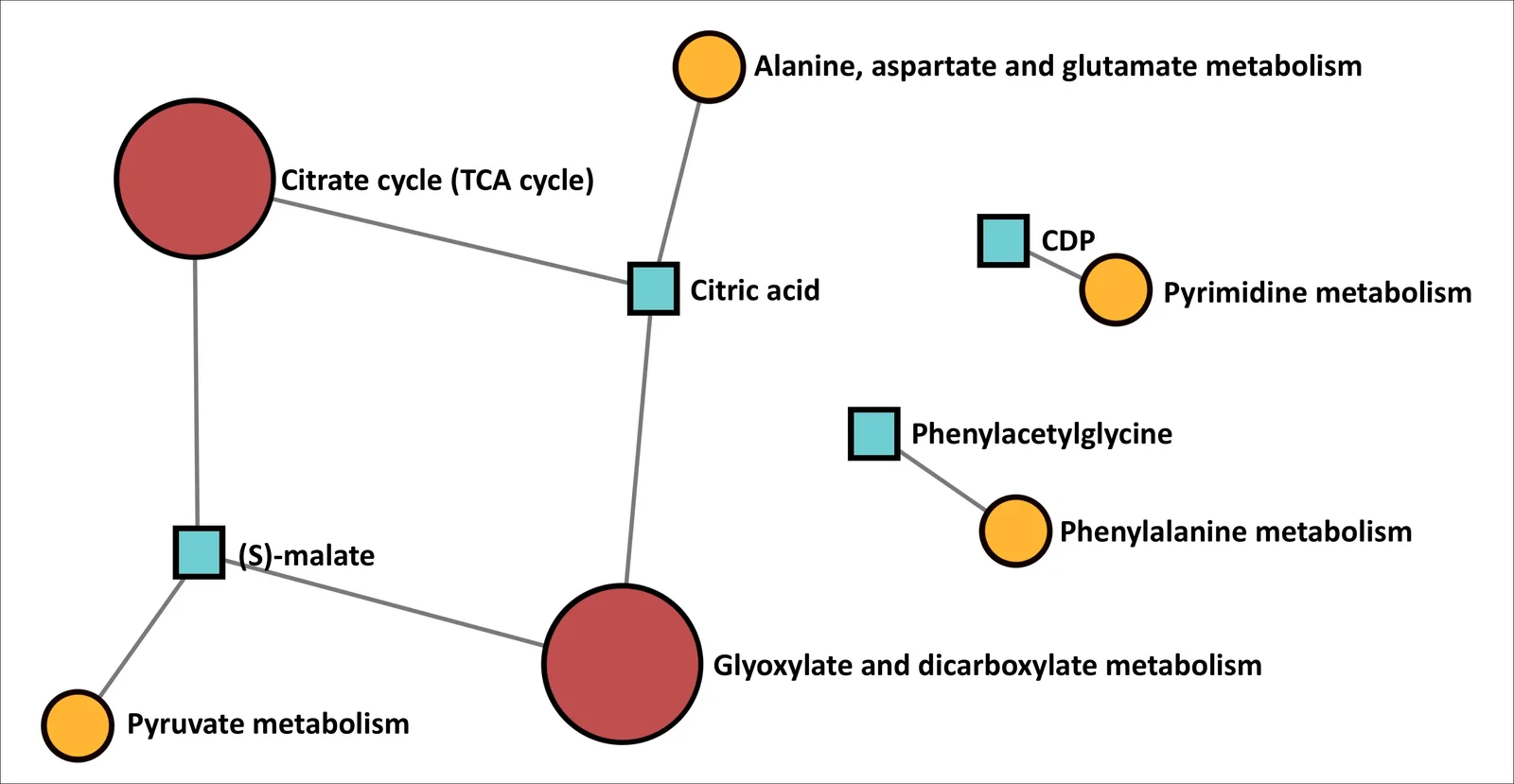
Metabolite-set enrichment network map comparing control and *Staphylococcus chromogenes*-infected quarters and illustrating the metabolic networks associated with the candidate metabolites. MetPA = Metabolic pathway analysis.

**Table 4:** Metabolic pathway analysis of candidate metabolites associated with S. chromogenes infection

**Pathway**	**Hits/total**	**Metabolite(s)**	**p-value**	**FDR**	**Impact**
Citrate cycle (TCA cycle)	2/20	Citric acid, (S)-malate	<0.01	0.07	0.14
Glyoxylate and dicarboxylate metabolism	2/32	Citric acid, (S)-malate	<0.01	0.09	0.08
Phenylalanine metabolism	1/10	Phenylacetylglycine	0.03	0.69	0.00
Pyruvate metabolism	1/23	(S)-malate	0.06	1.00	0.03
Alanine, aspartate, and glutamate metabolism	1/28	Citric acid	0.07	1.00	0.00
Pyrimidine metabolism	1/38	Cytidine 5′-diphosphate	0.09	1.00	0.02

FDR = False discovery rate; TCA = Tricarboxylic acid.

## DISCUSSION

Mastitis remains one of the most prevalent diseases affecting the dairy industry, with subclinical mastitis posing a particular challenge because infection may persist without visible abnormalities of the udder or milk. In the present study, all *S. chromogenes*-infected quarters were classified as subclinical, and SCC was significantly higher in infected than in control quarters. This finding supports SCC as a useful indicator of mammary inflammation; however, SCC lacks pathogen specificity and may also be influenced by parity, stage of lactation, and physiological stress [38, 39]. Therefore, SCC should be interpreted together with bacteriological culture and clinical findings. The increased SCC observed in *S. chromogenes*-infected quarters is consistent with an inflammatory response in the mammary gland despite the absence of clinical signs or visible milk abnormalities [40].

To our knowledge, this is the first untargeted milk metabolomic investigation focused specifically on naturally occurring *S. chromogenes* intramammary infection. The metabolomic analysis identified five candidate metabolites associated with infection status: citric acid, (S)-malate, cytidine 5′-diphosphate, acetylcarnitine, and phenylacetylglycine. At the nominal significance level, citric acid, (S)-malate, cytidine 5′-diphosphate, and phenylacetylglycine showed greater abundance in infected quarters, whereas acetylcarnitine showed lower abundance. Collectively, these changes suggest possible alterations in central energy metabolism, fatty-acid handling, amino acid metabolism, and nucleotide or membrane turnover. However, given the exploratory design, limited sample size, metabolite annotation confidence, and multiple-testing considerations, these findings should be interpreted as candidate metabolic signals rather than established biomarkers or definitive evidence of pathway dysregulation.

### Contrast with previous mastitis metabolomics

Metabolic profiling of milk from control and infected quarters, integrated with pathway analysis, indicated that several of the candidate changes involved central energy metabolism. In particular, the greater relative abundance of citric acid and (S)-malate, both intermediates of the TCA cycle [41], suggests altered mammary energy metabolism during *S. chromogenes* infection. TCA cycle involvement is broadly consistent with previous milk metabolomic studies in which central energy metabolism was among the pathways affected during intramammary infection [42–44].

Nevertheless, the direction of these changes differs from that reported in several previous studies. Xi *et al.* [43], using UPLC-Q-TOF-MS, reported lower citrate, L-carnitine, and hippurate concentrations in milk from cows with clinical mastitis than in healthy controls. Sundekilde *et al.* [42] similarly reported that increased SCC was associated with decreased fumarate and hippurate. A more recent quarter-level investigation interpreted decreases in citrate and cis-aconitate at the peak of infection as evidence of reduced TCA cycle activity [45]. In contrast, citric acid and (S)-malate showed greater relative abundance in *S. chromogenes*-infected quarters in the present study.

Several factors could potentially contribute to this difference. Previous investigations often examined clinical mastitis, major mastitis pathogens, or different infection stages, whereas the present study specifically evaluated naturally occurring, subclinical *S. chromogenes* infection. Metabolic responses may vary according to pathogen, infection stage, inflammatory intensity, and host physiological status, and pathogen-dependent differences in citrate metabolism have been reported [44]. The biological compartment analyzed may also influence the observed direction of metabolic changes because the milk-fluid metabolome does not necessarily reflect metabolic alterations in mammary tissue directly. Thus, the increased relative abundance of citric acid and (S)-malate observed here may represent a pathogen- or disease-stage-associated pattern. Nevertheless, targeted validation is required before this pattern can be considered characteristic of *S. chromogenes* subclinical mastitis.

### Biological interpretation of candidate metabolic changes

The increased relative abundance of citric acid and (S)-malate may reflect changes in host cellular energy metabolism during mammary inflammation. Recruitment and activation of immune cells increase cellular energetic and biosynthetic demands and altered oxidative metabolism may redirect citrate toward immunometabolic and lipid-biosynthetic processes [46]. This metabolic remodeling could contribute to changes in TCA cycle intermediate concentrations in milk. This interpretation is compatible with the increased SCC observed in infected quarters, which indicates leukocyte recruitment to the mammary gland.

Microbial metabolism could also contribute to the observed metabolomic profile. *Staphylococcus *species, including *Staphylococcus epidermidis*, can modulate TCA cycle activity in response to environmental stress, with potential consequences for bacterial survival and antimicrobial tolerance [47]. In host cells, TCA cycle activity is likewise tightly regulated, and its intermediates are altered in inflammatory and other disease states [48, 49]. Because the milk metabolome may contain metabolites from both host and microbial processes [43], the present data cannot distinguish the relative contributions of mammary epithelial cells, infiltrating leukocytes, and *S. chromogenes*. In addition, (S)-malate has been implicated in intracellular pH regulation and immune signaling in macrophages [50, 51]. Therefore, its increased abundance could potentially reflect inflammatory metabolic remodeling; however, this mechanism was not directly investigated in the present study.

Acetylcarnitine showed lower relative abundance in *S. chromogenes*-infected quarters. Although acetylcarnitine was not incorporated into MetPA because no corresponding annotated node was available in the *Bos taurus* KEGG pathway maps, this database limitation does not necessarily diminish its potential biological relevance [52, 53]. Acetylcarnitine participates in mitochondrial energy homeostasis and acetyl-group metabolism and contributes to regulation of the mitochondrial acetyl-CoA pool [54]. It has also been associated with anti-inflammatory and cytoprotective activities [54–56]. Its lower abundance in infected quarters may therefore be compatible with altered mitochondrial substrate utilization or inflammatory metabolic stress. Nevertheless, the present data cannot determine whether reduced acetylcarnitine resulted from increased utilization, reduced synthesis, altered transport, or another mechanism. Manual pathway curation, alternative pathway databases, and targeted quantitative analyses may help clarify its relevance to *S. chromogenes* infection.

Phenylacetylglycine showed greater relative abundance in infected quarters. This metabolite is associated with the metabolism of phenylacetic acid arising from phenylalanine-related metabolic processes [57–59]. Compared with citrate and carnitine-related metabolites, phenylacetylglycine has been reported less consistently in mastitis metabolomic studies. Its increased abundance could potentially reflect altered host protein and amino acid metabolism, microbial metabolism, or their interaction during inflammation. Li *et al.* [60] reported reduced phenylacetylglycine-related metabolism during mammary inflammation, which contrasts with the direction observed in the present study. This difference further suggests that metabolomic responses may vary according to pathogen, disease stage, and experimental context. Phenylacetylglycine should therefore be considered a candidate metabolic feature requiring pathogen-specific validation.

Cytidine 5′-diphosphate showed the largest VIP score among the five prioritized metabolites. Cytidine nucleotides participate in nucleotide metabolism and provide intermediates for membrane phospholipid biosynthesis through pathways involving CDP-choline and CDP-diacylglycerol [61, 62]. Its increased relative abundance may therefore be compatible with increased nucleotide and membrane turnover associated with inflammatory cell activity and tissue responses. However, because neither cellular proliferation nor membrane turnover was directly measured, this interpretation remains hypothetical. Moreover, the analytical uncertainty associated with this metabolite requires particular consideration before biological conclusions are drawn from its apparent increase.

### Analytical confidence and need for orthogonal validation

The biological interpretation of the candidate metabolites depends on the confidence of their analytical identification. The metabolites were assigned using the untargeted annotation workflow described in the Methods, and the individual LoA classifications should therefore be considered when interpreting the findings. Citric acid, (S)-malate, acetylcarnitine, and phenylacetylglycine were assigned LoA 3, whereas cytidine 5′-diphosphate was assigned LoA 2. Under the applied LoA framework, the LoA 3 assignments have greater analytical support than the LoA 2 assignment; however, none of these metabolites was validated against an authentic reference standard. Consequently, these compounds should not be regarded as unequivocally confirmed identities. Additional caution is warranted for specific analytical features. Citric acid was detected as a sodium adduct ([M+Na]⁺), and the possibility of adduct ambiguity or isobaric interference should therefore be considered. Similarly, the observed m/z of the cytidine 5′-diphosphate feature (404.03361) deviated by approximately +20 ppm from the theoretical [M+H]⁺ value (404.02546). Although this deviation was within the MS1 mass tolerance applied during annotation (0.01 Da), it was larger than, and opposite in direction to, those of the other candidate metabolites (approximately −6 to −13 ppm). Because targeted quantification, absolute concentration determination, and orthogonal confirmation using authentic standards were not performed, the five metabolites should presently be regarded as putatively annotated candidate features. Targeted MS/MS analysis and co-elution with authentic standards would provide substantially stronger evidence for metabolite identity and should be prioritized in subsequent studies.

### Exploratory status, multiplicity, and model performance

The metabolomic findings should be interpreted within the exploratory and hypothesis-generating framework of this study. Untargeted profiling generated a high-dimensional dataset relative to the number of biological samples, increasing the potential for chance associations. Candidate metabolites were prioritized using OPLS-DA-derived parameters followed by univariate testing. Consequently, nominal p-values alone are insufficient to establish robust differential abundance when multiple metabolites are evaluated simultaneously.

The pathway-level findings similarly require cautious interpretation. The TCA cycle, glyoxylate and dicarboxylate metabolism, and phenylalanine metabolism showed nominal enrichment, but none remained significant after FDR correction. Therefore, these pathways should be regarded as candidate biological processes potentially associated with *S. chromogenes* infection rather than statistically confirmed pathway alterations. Their biological coherence with the individual candidate metabolites nevertheless provides a basis for targeted investigation in larger datasets.

The OPLS-DA model showed R²X = 97.62%, R²Y = 96.89%, and Q² = 55.28%, with a permutation test p = 0.03. These results indicate a close fit to the present dataset but more moderate predictive performance. This permutation test assumed that quarters were exchangeable, whereas four of the nine cows contributed quarters to both groups; a within-cow restricted permutation that preserves this clustering admits only 24 distinct label assignments and yields an exact p of 0.042, the smallest value the design can produce. Given the small number of samples relative to the large metabolomic feature space, overfitting remains an important consideration despite the permutation test result. Accordingly, the observed separation should not be interpreted as evidence of established diagnostic discrimination. External validation using an independent dataset will be necessary to determine whether the identified metabolomic pattern is reproducible and predictive.

### Sample size and non-independence of observations

An important limitation of the study is the size and hierarchical structure of the dataset. Fifteen quarter-milk samples were obtained from nine cows, and four cows contributed both control and infected quarters, and sampling was unbalanced across the three participating farms. The 15 quarter-level observations were not fully independent. The limited sample size and unbalanced distribution of quarter infection status within individual cows precluded reliable estimation of cow- and farm-level random effects, and the analyses were consequently performed at the quarter level, treating quarters as independent observations. This approach may not fully account for within-cow correlation and may render permutation-based significance estimates anticonservative, as reflected in the restricted permutation result reported above.

Several potential sources of biological variation were nevertheless restricted through the study design. The study included primiparous cows in early lactation, and animals with clinical disease, lameness, recent medication use, or evidence of urinary ketones were excluded. In addition, the participating farms belonged to the same dairy cooperative and followed broadly comparable management practices. These measures reduced, but could not eliminate, potential confounding. The findings therefore require confirmation in a larger, independently recruited cohort that permits hierarchical modeling of cow- and farm-level effects.

### Implications and future directions

Despite these limitations, the pathogen-specific design represents an important feature of the present study. Many previous mastitis metabolomic investigations have evaluated major pathogens, mixed infections, or etiologically heterogeneous mastitis populations. Restricting the analysis to microbiologically confirmed *S. chromogenes* infection allowed the identification of candidate metabolic changes associated with this specific form of subclinical mastitis. The convergence of the prioritized metabolites on central energy metabolism, carnitine-associated metabolism, phenylalanine-related metabolism, and nucleotide or membrane metabolism provides biologically plausible hypotheses for subsequent investigation.

Future studies should first confirm the identities and absolute concentrations of citric acid, (S)-malate, cytidine 5′-diphosphate, acetylcarnitine, and phenylacetylglycine using targeted LC-MS/MS with authentic standards. These candidates should then be evaluated in larger, multi-farm cohorts with sufficient numbers of cows to account statistically for quarter-level clustering within animals and farm-level variation. Comparative analysis of *S. chromogenes* with other CNS species and major mastitis pathogens would help determine whether the observed metabolomic pattern is pathogen-specific or represents a broader response to subclinical intramammary infection. Integration of metabolomic data with transcriptomic or proteomic profiling could further clarify the cellular pathways underlying these candidate metabolic alterations and distinguish host-derived responses from microbial contributions.

## CONCLUSION

This exploratory study provides pathogen-specific evidence that naturally occurring *S. chromogenes* associated subclinical mastitis is accompanied by measurable alterations in the milk metabolomic profile of early lactation dairy cows. Infected quarters had significantly higher SCC than control quarters despite the absence of clinical mastitis, supporting the presence of an active subclinical mammary inflammatory response. Untargeted metabolomic profiling identified five candidate metabolites associated with infection status. Citric acid, (S)-malate, cytidine 5′-diphosphate, and phenylacetylglycine showed greater relative abundance in infected quarters, whereas acetylcarnitine showed lower abundance. OPLS-DA showed separation between groups, with good model fit but moderate predictive performance, while pathway analysis implicated the TCA cycle, glyoxylate and dicarboxylate metabolism, and phenylalanine metabolism at the nominal significance level. Collectively, these findings suggest that *S. chromogenes* infection may be associated with coordinated changes in mammary energy, amino acid, fatty-acid, and nucleotide-related metabolism.

A major strength of this study is its pathogen-specific design based on naturally occurring, microbiologically confirmed *S. chromogenes* intramammary infection rather than mixed-pathogen mastitis. The use of quarter-level sampling, stringent case and control definitions, dual-polarity untargeted metabolomics, systematic QC procedures, and complementary multivariate and pathway analyses provide a useful framework for identifying metabolic responses associated with this frequently encountered cause of subclinical mastitis. Nevertheless, the findings should be interpreted as hypothesis-generating because of the small sample size, partial within-cow dependence of observations, limited representation of farms and animals, absence of external validation, and uncertainty inherent in putative metabolite annotation. In addition, the pathway-level associations did not remain significant after FDR correction, and the metabolite identities and relative abundance differences require confirmation by targeted quantitative methods.

Overall, this study provides preliminary evidence of a distinct candidate milk metabolic profile associated with *S. chromogenes* subclinical mastitis and establishes a foundation for pathogen-specific metabolomic investigation of bovine intramammary infection. Future studies should validate the prioritized metabolites using targeted LC-MS/MS and authentic standards in larger, independent, multi-farm cohorts with appropriate hierarchical statistical modeling and should compare *S. chromogenes* with other mastitis pathogens to determine the specificity of the observed metabolic pattern. Integration of metabolomics with transcriptomic or proteomic approaches may further clarify the biological origins of these alterations. Such validation will determine whether these candidate metabolic signatures can ultimately contribute to a more mechanistic understanding and improved detection of pathogen-specific subclinical mastitis.

## DATA AVAILABILITY

The raw LC-MS metabolomics data generated in this study are publicly available in the MassIVE repository under accession number MSV000102009 (https://doi.org/10.25345/C5K35MT79). All other data supporting the findings of this study are included in the article and its Supplementary Information files.

## GENERATIVE AI DECLARATION

The authors declare that generative artificial intelligence (AI) tools were used solely to improve language, grammar, and readability during manuscript preparation. All scientific content, data analysis, interpretation of results, and conclusions were developed and verified by the authors. The authors take full responsibility for the accuracy, integrity, and originality of the work presented, and no AI tool was listed as an author.

## AUTHORS’ CONTRIBUTIONS

SS and SR: Conceptualization. SS and SR: Methodology. SS and SR: Validation. SS: Formal analysis. SS and SR: Investigation. SS and SR: Resources. SS: Data curation. SS: Writing – original draft. SS and SR: Writing – review and editing. SS: Visualization. SR and SS: Supervision. SS: Project administration. SR: Funding acquisition. All authors have read and approved the final version of the manuscript.
